# HeartMate 3 Implantation Through Left Atrial e-PTFE Conduit for Restrictive Cardiomyopathy

**DOI:** 10.1016/j.atssr.2022.10.015

**Published:** 2022-10-29

**Authors:** Aaron Guo, Melanie P. Subramanian, Justin Vader, Kory Lavine, Joel Schilling, Justin Hartupee, Kunal Kotkar, Akinobu Itoh

**Affiliations:** 1 Division of Cardiothoracic Surgery, Department of Surgery, Washington University in St Louis School of Medicine, St Louis, Missouri; 2 Division of Cardiovascular Diseases, Department of Medicine, Washington University School of Medicine in St Louis, St Louis, Missouri; 3 Department of Pathology and Immunology, Washington University School of Medicine in St Louis, St Louis, Missouri; 4 Division of Thoracic and Cardiac Surgery, Department of Surgery, Brigham and Women's Hospital, Harvard Medical School, Boston, Massachusetts

## Abstract

Restrictive or hypertrophic cardiomyopathy presents a challenge to left ventricular assist device placement because of the small left ventricle cavity. Cases have described inflow cannulation of the HeartWare HVAD by expanded polytetrafluoroethylene conduit through the atrial septum to the left atrium. We applied this technique to an adult man with restrictive cardiomyopathy and pulmonary hypertension using the HeartMate 3, which successfully supported the patient and led to significant reduction in pulmonary artery pressure. He received a transplant 3 months later without complications. For select patients, left atrial conduit for HeartMate 3 inflow is a feasible alternative to conventional apical cannulation.

Patients with small left ventricular (LV) cavities secondary to restrictive or hypertrophic cardiomyopathy are problem candidates for left ventricular assist device (LVAD) placement because of obstruction of inflow.^1^^,^^2^ Maeda and colleagues^3^ described placement of a HeartWare HVAD (Medtronic) connected to an expanded polytetrafluoroethylene (e-PTFE) conduit tunneled through the right atrium, sewn to the fossa ovalis in a pediatric patient. Kiamanesh and associates^2^ successfully replicated this procedure in a 57-year-old man. However, HVAD was recently discontinued, and the HeartMate 3 (HM3; Abbott) is the only available LVAD in the United States. We report a case of an adult with restrictive cardiomyopathy who was a poor candidate for transplantation because of severe pulmonary hypertension (pHTN), for whom this technique was employed with an HM3 device.

We present a 57-year-old man with chronic heart failure due to nonischemic restrictive cardiomyopathy and severe pHTN (pulmonary artery [PA] pressure, 100/52 mm Hg; systemic pressure, 109/47 mm Hg; right atrial [RA] pressure, 22 mm Hg; pulmonary capillary wedge pressure, 32 mm Hg; and pulmonary vascular resistance [PVR], 6.0 Wood units). He was prescribed milrinone and bumetanide infusions and inhaled nitric oxide. LV end-diastolic and systolic dimensions were 42 mm and 15 mm, respectively. Because of progressive heart failure and worsening end-organ perfusion, he was supported by an intra-aortic balloon pump. Given his deconditioning and pHTN, he was deemed not to be a candidate for direct transplantation. Because of his LV anatomy, conventional LVAD was also not possible. Therefore, the nonconventional left atrial (LA) conduit HM3 was pursued as his only treatment option.

Through median sternotomy, cardiopulmonary bypass was established with aortic and bicaval cannulations. The right atrium was opened longitudinally 2 cm from the atrioventricular groove. With the heart arrested, the fossa ovalis was resected and enlarged, to which a 7-cm-long, 20-mm e-PTFE ring-reinforced conduit (W. L. Gore & Associates) was sewn with running 4-0 monofilament sutures. The HM3 inflow cannula was inserted into the other end of the conduit and secured with a cable tie (Figure 1). The atriotomy was closed tightly around the conduit. The outflow graft was anastomosed to the ascending aorta (Figure 2) without bend relief. He was weaned off cardiopulmonary bypass uneventfully. The LVAD was affixed to the right chest wall with a large suture around the sixth rib (Figure 3). A Gore-Tex membrane was placed around the device to prevent it from contacting the right lung and superior vena cava. The patient was supported successfully with this configuration and discharged home 3 weeks postoperatively with LVAD settings of 5400 rpm yielding 4.5 L/min and pulsatility index of 3.0. Mean arterial pressure was 76 mm Hg, and prothrombin time/international normalized ratio was 2.7. Eight weeks later, right-sided heart catheterization showed PA pressure of 37/11 mm Hg, systemic pressure of 106/85 mm Hg, RA pressure of 13 mm Hg, and pulmonary capillary wedge pressure of 13 mm Hg with PVR of 2.0 Wood units.

**Figure 1:**
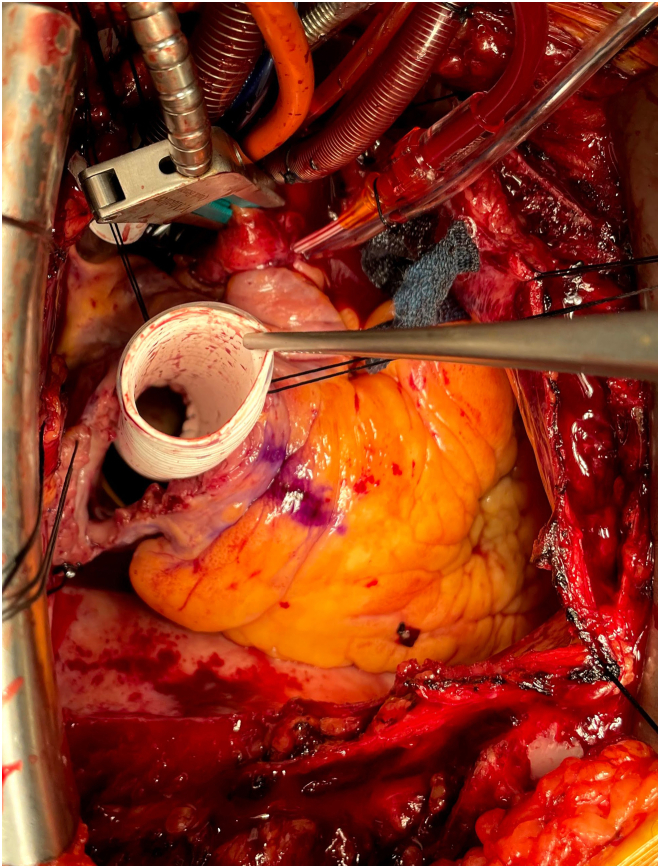
Ring-reinforced expanded polytetrafluoroethylene conduit sewn to the fossa ovalis.

**Figure 2:**
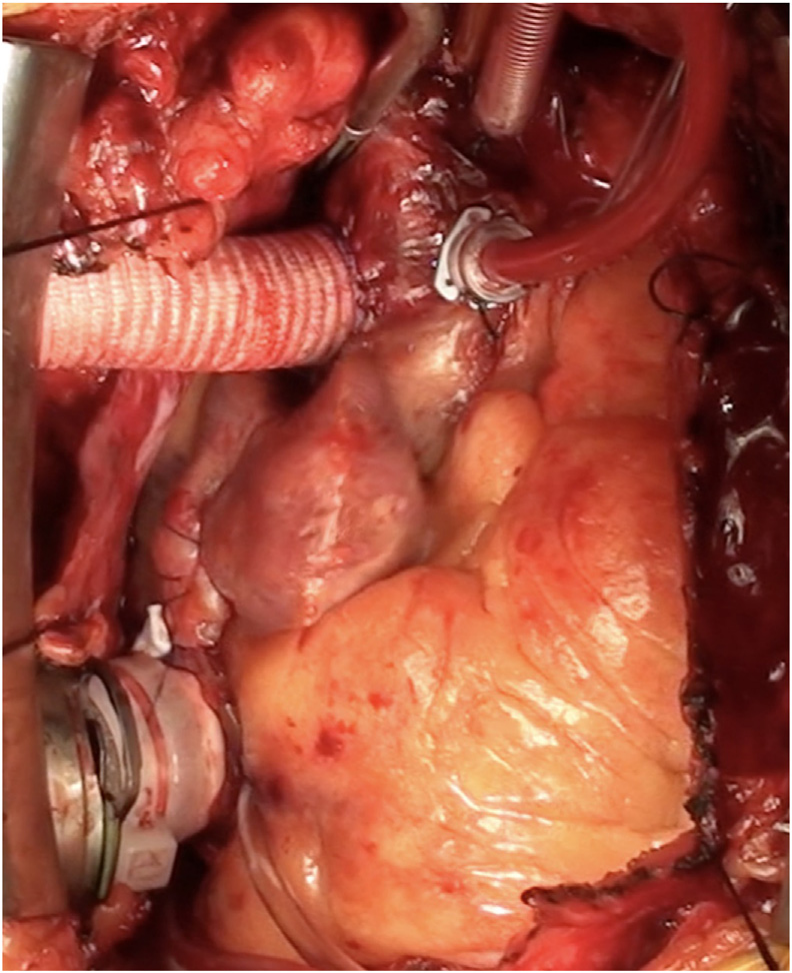
HeartMate 3 residing over the right atrium.

**Figure 3:**
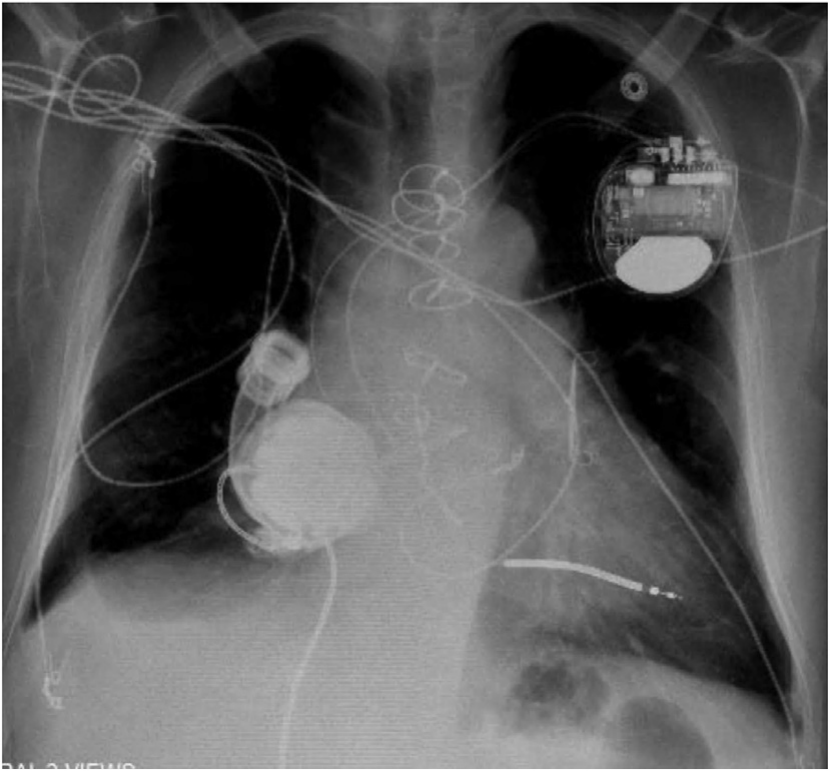
Chest radiograph demonstrates HeartMate 3 positioning in the right side of the chest.

After 3 months, his PA pressures and PVR had decreased significantly, and he underwent heart transplantation. Chest entry and dissection of the LVAD were straightforward (Supplemental Figure 1). The inflow conduit was excised from the atrial septum, and the subsequent defect was incorporated in the LA anastomosis suture line. No thrombus was seen in the explanted heart. He was discharged home 2 weeks after transplantation. LV ejection fraction was 67% at 9 months’ follow-up.

## Comment

Maeda and colleagues^3^ were the first to employ an LA conduit configuration in a pediatric patient using the HVAD, followed shortly by Kiamanesh and associates^2^ in an adult patient. Building on these efforts, we have applied the technique with the HM3, which is currently the only Food and Drug Administration–approved commercially available LVAD. Most patients with restrictive or hypertrophic cardiomyopathy are unsuitable for conventional LVAD placement because their small LV cavity cannot accommodate the inflow cannula, increasing risk of suction events and low-flow alarms. Other alternatives to conventional LVAD placement have been described, such as LV myomectomy before apical cannulation or biventricular assist device placement.^4^, ^5^, ^6^ As presented here, sufficient inflow can be achieved by draining the left atrium through the interatrial conduit. Furthermore, compared with these other methods, we believe that the LA conduit technique simplifies the operation with good exposure through the right atrium while limiting additional interventions.

Differences between the HM3 and HVAD were noted that are important considerations for operative planning. First, positioning of the HM3 pump is less forgiving because of the 60% larger volume and sharper edges of the housing compared with HVAD (Supplemental Figure 2). The atriotomy should not be too anterior or superior to prevent the device from compressing the right atrium. Preoperative assessment of RA size on computed tomography imaging is also critical to determine conduit length before the surgical procedure because distance from the atrial septum to the RA surface cannot be estimated once the right atrium is decompressed. Second, the tip of the HM3 inflow cannula is sharply cylindrical without bevel, whereas that of the HVAD is smoothly rounded, although both are 20 mm in diameter. Furthermore, the surface of the HM3 inflow cannula is entirely sintered, providing a much rougher texture relative to the HVAD, which is sintered only in the middle of the cannula (Supplemental Figure 2). These aspects of the HM3 led to difficulty in inserting the cannula into the e-PTFE conduit, particularly as both should be the same diameter. To facilitate this cumbersome process, we recommend stretching the conduit before inserting the LVAD. Finally, the ring-enforced conduit is difficult to manipulate during anastomosis in the limited space of the right atrium; it is advantageous to use long continuous sutures and to parachute the entire apparatus onto the atrial septum.

The main drawback of LA drainage is decreased flow through the left ventricle. Particularly at higher LVAD flow rates, this may divert too much blood flow and increase risk of LV stagnation and intraventricular or aortic root thrombus. For this reason, we targeted a higher international normalized ratio goal of 2.5 to 3.5 and focused on allowing the aortic valve to open every few ejections. For select patients with restrictive or hypertrophic cardiomyopathy, HM3 inflow by e-PTFE conduit to the left atrium can be a reasonable approach to reduce pHTN and to provide adequate support without inflow restrictions.
