# Supplementary material for: HeartMate 3 Implantation Through Left Atrial e-PTFE Conduit for Restrictive Cardiomyopathy
Source: Ann Thorac Surg Short Rep. 2022 Oct 29;1(1):191–3. doi: 10.1016/j.atssr.2022.10.015 (PMC11708496; doi:10.1016/j.atssr.2022.10.015)

**Supplemental Figure 1**: HeartMate3 LVAD in right chest at time of transplantation is easily separated from anterior chest wall and right lung due to Gore-Tex membrane
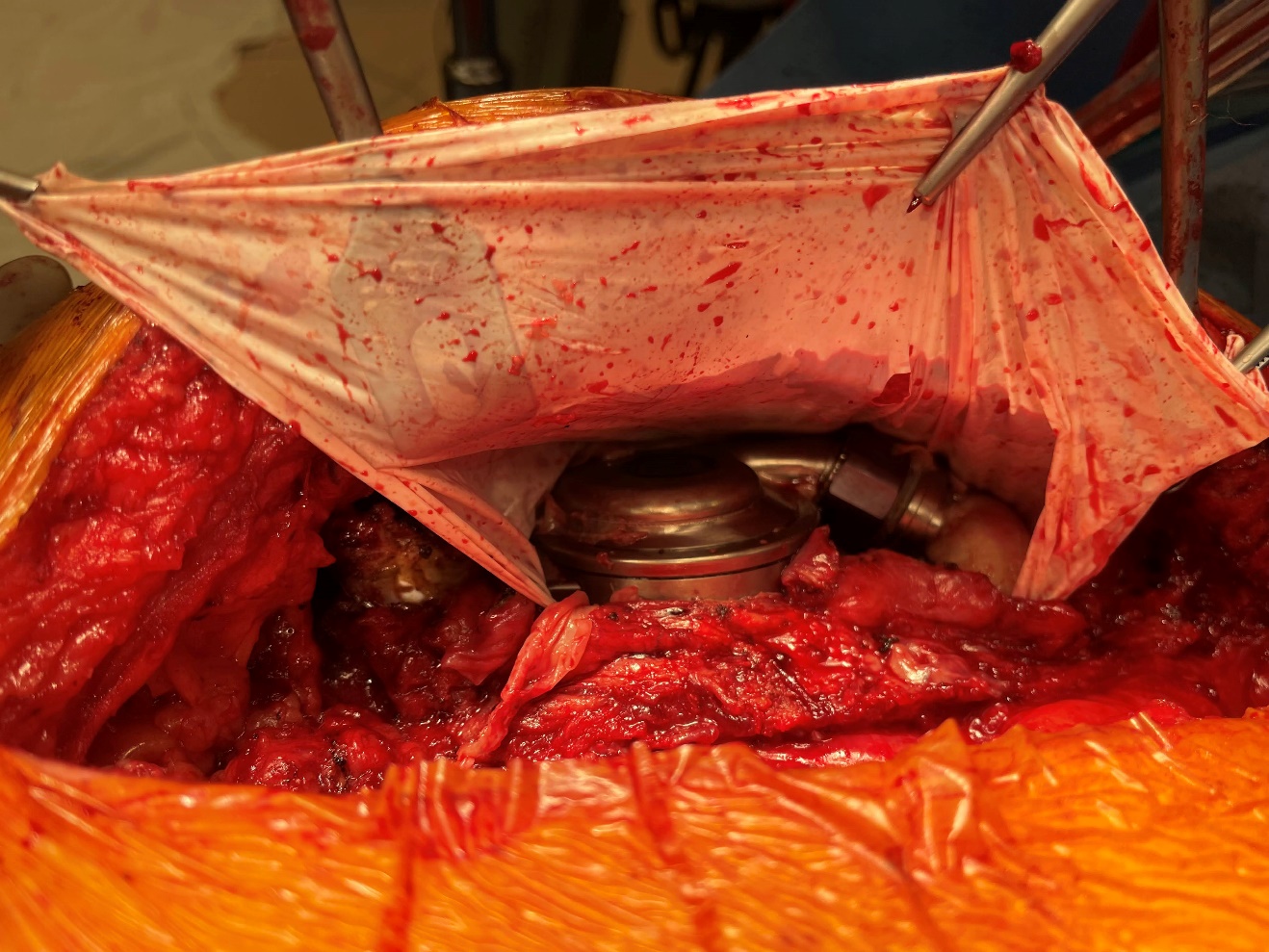


**Supplemental Figure 2.** Comparison of hVAD (left) and HeartMate3 (right) devices shows the larger size and sharper edges of the HeartMate3 housing. The HeartMate3 inflow cannula is the same diameter (20 mm) but sintered along the entire length and with a cylindrical, non-beveled tip.


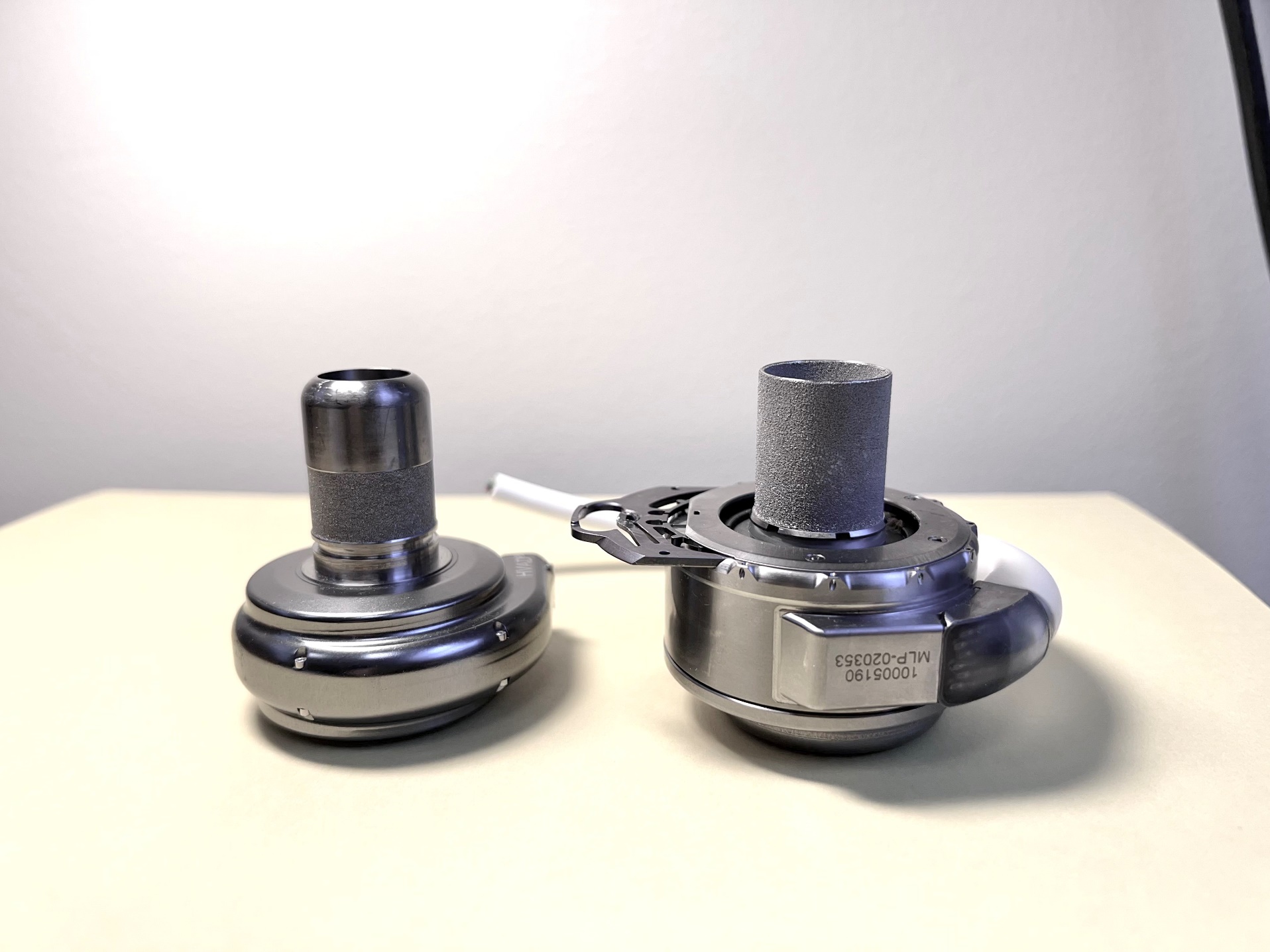

Supplement: Supplementary Figures [file mmc1.docx]
